# A modular platform for measuring substrate oxidation by CO_2_ production in diverse cell culture systems

**DOI:** 10.1016/j.jbc.2026.113384

**Published:** 2026-08-04

**Authors:** James R. Krycer, Shubhangi Seth, Lynn A.C. Devilée, Mary Lor, Rebecca L. Fitzsimmons, Richard J. Mills, Yan Lu, Prarthna G. Bhaskaran, Mark Hodson, James E. Hudson, Severine Navarro

**Affiliations:** 1 QIMR Berghofer, Brisbane, Queensland, Australia; 2 School of Biomedical Sciences, Faculty of Health, Queensland University of Technology, Brisbane, Queensland, Australia; 3 Murdoch Children's Research Institute, The Royal Children's Hospital, Melbourne, Victoria, Australia; 4 Novo Nordisk Foundation Center for Stem Cell Medicine, Murdoch Children's Research Institute, Melbourne, Victoria, Australia; 5 Department of Paediatrics, University of Melbourne, Melbourne, Victoria, Australia; 6 School of Biomedical Sciences, Faculty of Health, Medicine and Behavioural Sciences, The University of Queensland, Brisbane, Queensland, Australia; 7 School of Pharmacy, Faculty of Health, Medicine and Behavioural Sciences, The University of Queensland, Brisbane, Queensland, Australia

**Keywords:** cell metabolism, dendritic cell, fatty acid metabolism, gas trap, glucose metabolism, immunometabolism, organoid, substrate oxidation

## Abstract

Metabolism underpins cellular physiology, whereby the preference for specific substrates and catabolic pathways shapes the production of energy, anabolic substrates, and metabolite signals to address bioenergetic demands. Substrate catabolism can be directly examined by measuring metabolic endpoints. For instance, substrate oxidation can be quantified by the incorporation of carbon from labelled glucose or fatty acids into carbon dioxide, providing a sensitive and specific readout of metabolic flux. However, current platforms require relatively large culture volumes, lacking adaptability for small-scale or complex cell culture formats. Herein, we develop and validate a modular platform that can quantify substrate oxidation in a range of cell culture systems, including two- and three-dimensional cultures grown in 12- and 96-well plate formats. This platform was engineered for precise gas equilibration, minimal gas leakage, and bioinert adapters suitable for smaller-scale cultures, using inexpensive and accessible components. We demonstrate the versatility of this system by showing that: (*i*) dendritic cells modulate glucose catabolism in response to a tolerance-inducing biologic (anti-inflammatory protein 2), and (*ii*) human cardiac organoids maintain fatty acid oxidation during acute inflammatory stress. This platform can be performed in parallel with orthogonal metabolomics assays and live-cell imaging, enabling integrated analysis of metabolic and functional readouts. Together, this platform expands access to measuring substrate oxidation across a range of cellular systems.

Cellular physiology is underpinned by metabolism, whereby a combination of catabolic pathways provides the required energy, reducing power, and anabolic precursors. Metabolic dysfunction has been commonly observed in many chronic diseases, including cardiometabolic disease and inflammatory disorders (1, 2). This has sparked great interest in quantifying substrate catabolism across a range of biological contexts.

A major catabolic fate, substrate oxidation, can be directly measured by assaying the incorporation of isotopically labelled substrates into CO_2_ (reviewed in (3)). Briefly, cells or tissues are incubated with ^14^C-labelled substrate (*e*.*g*., [U-^14^C]-glucose or [1-^14^C]-palmitate), which is metabolized to generate ^14^C-CO_2_. Cellular metabolism is quenched by acidification of the culture, releasing CO_2_ that is then trapped with an alkali solution (Fig. 1*A*). The radioactivity of the trapping solution (*i*.*e*., ^14^C-CO_2_ content) is then measured as a direct readout of substrate oxidation. Compared to alternatives such as respirometry (4) or ^13^C-flux analysis (5), this approach carries several advantages: (*i*) high sensitivity (often down to sub-nmol levels) and straight-forward detection by liquid scintillation counting; (*ii*) the ability to measure the oxidation of individual substrates even when multiple substrates are present, enabling the quantification of substrate competition (6) whilst avoiding indirect ‘subtractive’ approaches commonly used in respirometry; (*iii*) by controlling the duration of ^14^C-labelling, this assay provides a simple endpoint measurement under either steady and non-steady (dynamic) state conditions without requiring instrument equilibration.

**Figure 1:**
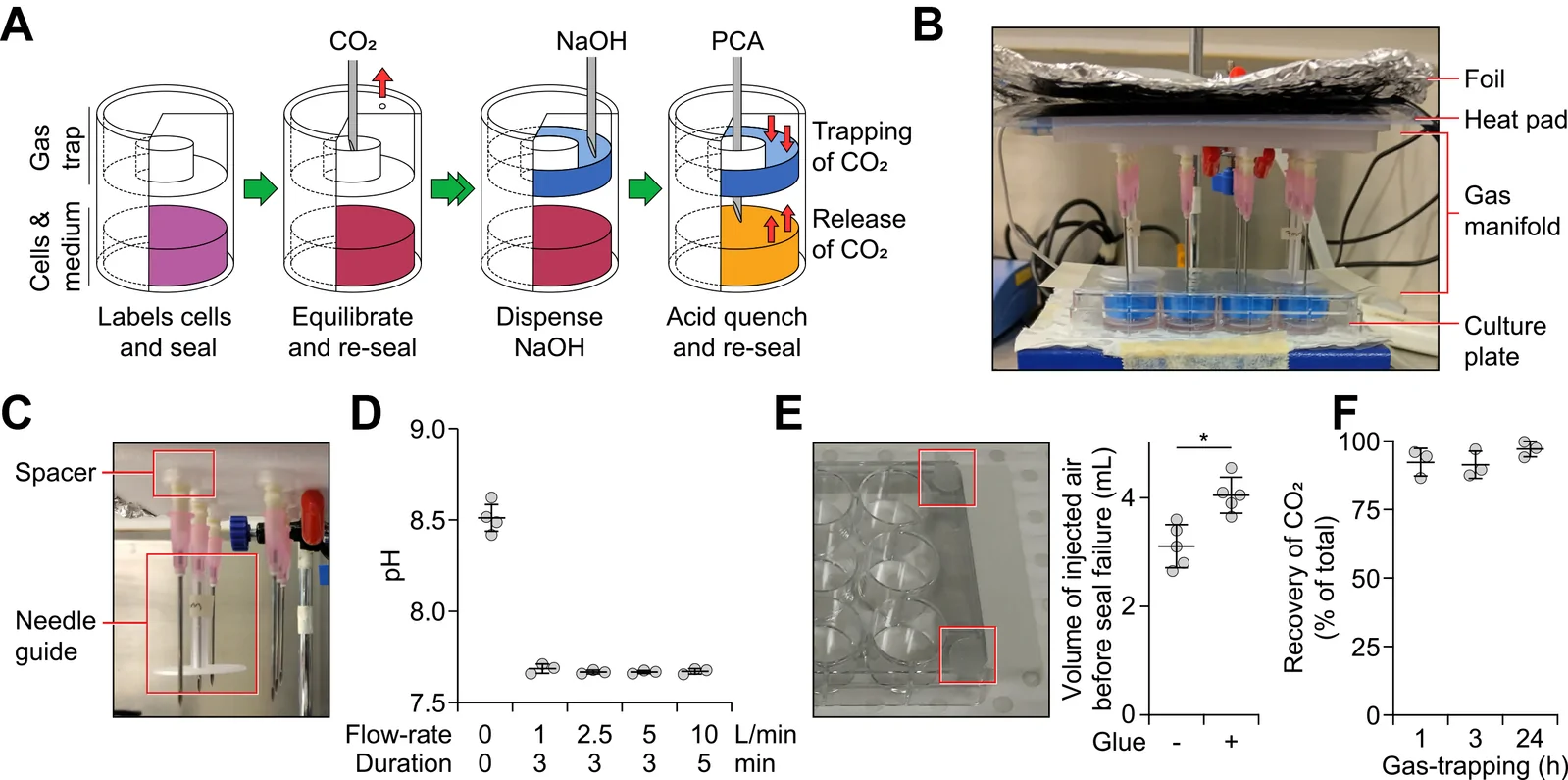
**Refinement of the substrate oxidation platform for miniaturized cultures.***A*, schematic of the general principle of the substrate oxidation assay. Details in the *Introduction* and Results. Adapted from (3). *B* and *C*, photographs depicting the anatomy of the gas manifold, with the installation of new spacers and needles guides (*C*). Details in Results. Close-up pictures of the needle guide are shown in Figure S1, *A* and *B*. *D*, culture media was equilibrated using the gas manifold (*B*) at the indicated flow-rates and durations, and pH was measured by phenol red absorbance, as described in the Experimental procedures. Data presented as mean ± *S.D*. from three separate experiments. *E*, *left*, Photograph depicting the position of the glue used to improve plate seal adherence. Details in Results. *Right*, evaluation of the impact of glue on plate seal adherence by air injection, as described in the Experimental procedures. Data presented as mean ± *S.D.*, *n* = 5 per condition. ∗*p* < 0.05, by *t* test. *F*, naïve culture media (Medium B) was preequilibrated with 5% (v/v) CO_2_ and labelled with 0.33 μCi/ml [^14^C]-NaHCO_3_ before being dispensed into 12-well plates and equilibrated with 5% (v/v) CO_2_ at 2.5 L/min for 3 min. After 1 h incubation at 37 °C, gas trapping solution (2 M NaOH) was dispensed, media was acidified, and plates re-sealed. At the indicated times, the gas trapping solution was collected and subjected to liquid scintillation counting. Radioactivity is expressed relative to the total radioactivity of the medium. Data presented as mean ± *S.D*., from three separate experiments.

Substrate oxidation assays have been a cornerstone of metabolic research over the past century. The original design involved culturing cells or tissues in a flask with the alkali solution housed in a central or hanging well (*e*.*g*., (7)). This is not amenable to the multiwell plate format used in modern tissue culture; thus, we previously adapted this assay for multiwell plates (8, 9). Here, cells are cultured at the bottom of each well and the alkali solution is housed in perforated tube caps inserted at the top of each well. We validated this methodology in both adherent and suspension cell cultures (9), generating flux rates directly comparable to those derived from respirometry (10) and ^13^C-tracer metabolic flux analysis (11, 12). This for instance revealed that adipocytes primarily rely on glucose fermentation rather than glucose oxidation (11) – in fact, glucose oxidation *via* the Krebs cycle and pentose phosphate pathway only made a minor contribution (∼20%) to total CO_2_ production (10).

We subsequently modified this assay to accommodate bicarbonate-buffered culture media, demonstrating that bicarbonate not only serves as a pH buffer, but also influences cell metabolism (3). This required engineering precise control over the timing of radiotracer labelling and gas-trapping following the addition of labelled substrate, culture plates were sealed to permit gas equilibration using a custom-built manifold, and alkali solution was only present for gas trapping after acidification. This platform was constructed from low-cost, readily available components, such as 3D-printed parts, standard laboratory reagents, and a reptile heating pad, to ensure wide accessibility.

With this philosophy in mind, here we addressed the increasing trend for miniaturization in tissue culture systems, developing a substrate oxidation assay suited for small-scale cultures. This required substantial modification of the gas equilibration system to improve precision and efficiency, and the development of novel, bio-inert adapters compatible with 96-well culture formats. This platform is now modular and compatible with diverse cell types, including cultured cells and organoids.

This platform complements existing metabolic methodologies. For instance, respirometry provides valuable information regarding cellular oxygen consumption and respiratory capacity, while stable-isotope flux analysis can provide broader insight into metabolic network activity. In contrast, the present assay directly quantifies the oxidation of specific substrates through their conversion to CO_2_, enabling substrate-specific measurements under conditions where multiple competing substrates are present. Thus, these approaches provide distinct, complementary information and can provide a holistic view of metabolism when used in combination (*e*.*g*., (10)).

## Results

### Refinement of the existing gas-trapping assay for miniaturized cultures

We previously developed a gas-trapping assay for cells cultured with bicarbonate-buffered media in a 12-well microplate format (3). Our central goal here was to adapt this protocol for small-scale cultures. One option involved using plates with smaller wells, such as replacing the 12-well plate with a 96-well plate to capture CO_2_ (13). However, this approach would have substantially increased handling time, particularly with the injection of alkali and perchloric acid (Fig. 1*A*), and required the manufacture of a 96-port gas manifold. Thus, we retained the 12-well plate format, instead aiming to culture cells in a smaller-diameter chamber inside each well. If more than 12 wells were required, the experiment could be performed with multiple plates, with appropriate controls and replicates spread appropriately across plates.

We initially focused on ensuring our original manifold design was compatible with this modified plate format (Fig. 1*B*). For instance, gas-equilibrating media within a smaller area of each well necessitated more precise control over the angle and depth of the outlet needles. To address this, we first incorporated 3D-printed spacers between the luer-slip connectors and manifold outlet ports (Fig. 1*C*), which minimized variability in the angle and depth of the adapters and thus needles attached to the manifold. Second, we designed and installed 3D-printed needle guides to precisely control the depth of needle insertion into the wells (Figs. 1*C* and S1, *A* and *B*). This prevented the needles from being inserted too deeply and accidentally contacting the culture media. Third, we reprinted the manifold with a higher infill percentage and thicker top and bottom layers (Table S2), to reduce gas leakage through the manifold walls. Evaluation of the updated manifold demonstrated that gas-equilibration could be achieved with a flow rate of 2.5 L/min for only 3 min (Fig. 1*D*). This reflects a substantial improvement over the previous version (3), which required 10 L/min for 5 min— that is, a ∼7-fold reduction in gas consumption.

Further, long-term use of our gas-trapping protocol (14) revealed the plate seal as a critical point of failure. Inadequate sealing enables CO_2_ to escape, which would alkalinize the bicarbonate-buffered media and lead to an underestimation of substrate oxidation. To minimize leakage, plate seals are applied before equilibration and additional layers of plate seals are added each time the seal is punctured by a needle. Nonetheless, we observed that the seal over the corner wells often became distorted after puncture, likely due to insufficient surface area at the plate corners for effective adhesion. To address this, we applied hot glue at the plate corners before sealing, shaping the glue to the desired height using a plastic scraper to improve seal integrity (Fig. 1*E*, *left*). This markedly reduced seal failure with the corner wells, evaluated by leakage after overnight inversion (3 *versus* 6 successfully sealed plates, no glue *versus* glue respectively, *n* = 6 plates per condition) and resistance to seal delamination with air injection (Fig. 1*E*, *right*).

Using this revised protocol, we assessed the recovery of ^14^C-labelled CO_2_. Cell-free media was labelled with ^14^C-NaHCO_3_, gas-equilibrated, and acidified. Using this optimized setup, >90% of the released CO_2_ was captured within an hour (Fig. 1*F*), supporting the effectiveness of plate seals in CO_2_-trapping assays. Having established the performance of the optimized gas-trapping platform, we next evaluated its utility in a biological system.

### Characterization of the metabolic response of dendritic cells using the optimized assay

We validated these modifications to the platform using cultured immune cells. We previously demonstrated that anti-inflammatory protein 2 (AIP-2), a hookworm-derived protein, exerts immune tolerance by acting upon dendritic cells (DCs) *in vivo* (15). Tolerogenic DCs exhibit increased mitochondrial respiration and glucose fermentation compared to immature DCs (16) and pro-inflammatory DCs (17). Thus, we employed our assay to determine how acute stimulation with AIP-2 impacted glucose utilization.

**Figure 2:**
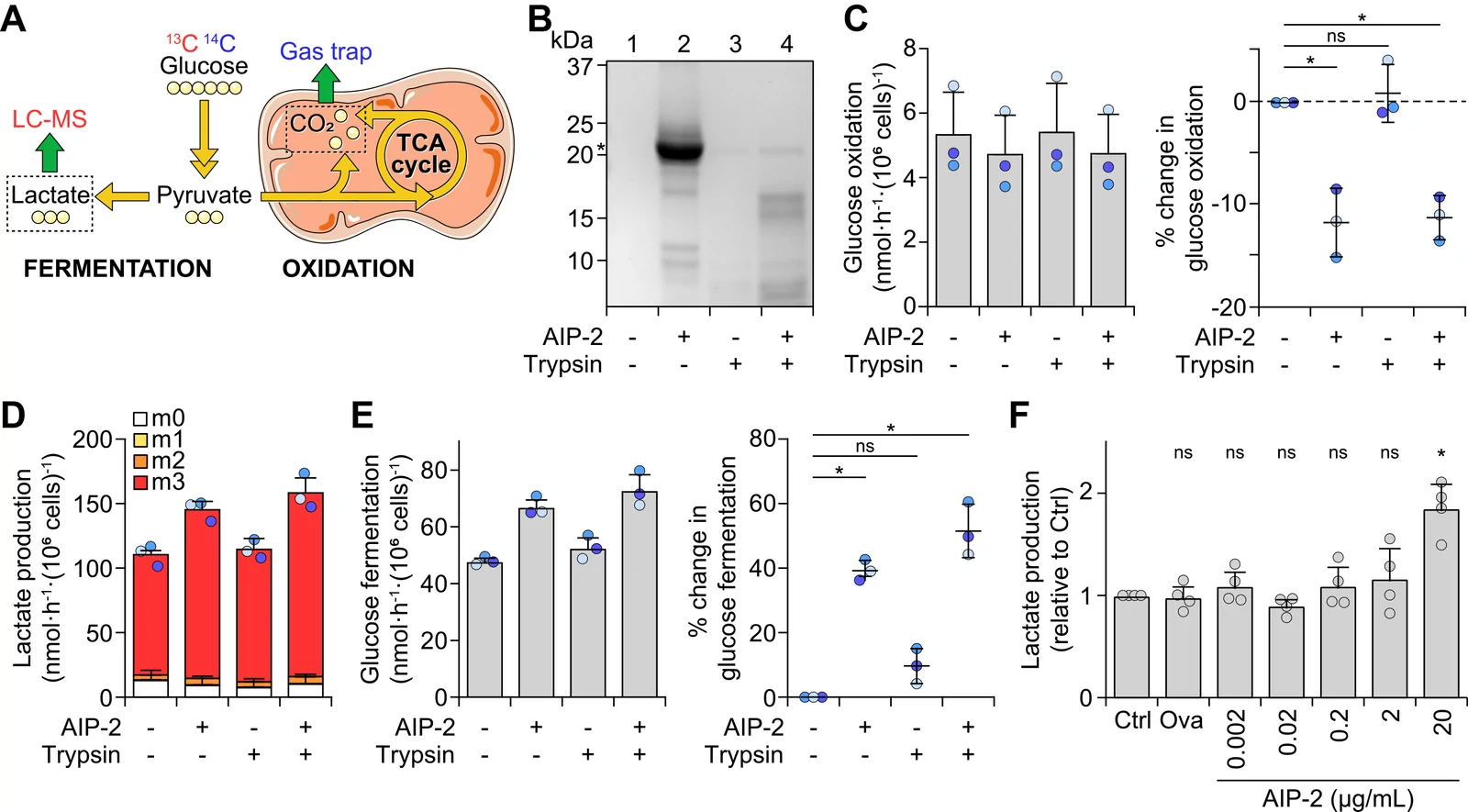
**AIP-2 modulates glucose catabolism in dendritic cells.***A*, schematic of how the major catabolic fates of glucose can be determined by isotopic labelling. For simplicity, this diagram excludes glucose oxidation through non-mitochondrial pathways such as the pentose phosphate pathway. Additional details in the Results. *LC-MS,* liquid chromatography coupled mass spectrometry. *B*, AIP-2 was digested with trypsin as outlined in the Experimental procedures, and subjected to SDS-PAGE and Coomassie staining. The expected size of full-length AIP-2 (20.4 kDa) is denoted by the *asterisk* (*∗*). Representative of four separate experiments. Note that trypsin co-elutes with AIP-2, as evident in Lane 3 (where AIP-2 is absent). Densitometric analysis indicated that only trace amounts of intact AIP-2 remained following trypsin digestion (0.050 ± 0.076%, mean ± *S.D.*, *n* = 4). *C*, mutuDC1940 cells were treated with intact or digested AIP-2, as prepared in *B* (*i*.*e*., PBS, trypsin, AIP-2, AIP-2 with trypsin), in the presence of [U-^14^C]-glucose and assayed for substrate oxidation as outlined in the Experimental procedures. Glucose oxidation is presented as the absolute amount of glucose converted into CO_2_ (*C*, *left*) and relative to the Ctrl condition (no AIP-2, no trypsin), that is percentage change whereby for instance “-10%” reflects a 10% decrease compared to the Ctrl condition (*C*, *right*). The latter shows that AIP-2 has a consistent effect on glucose oxidation despite variability in the baseline oxidation rate between experiments. Data presented as mean ± *S.D.* from 3 separate experiments, with each *dot color* denoting the data from each respective experiment. ∗, *p* < 0.05; ns, not significant (*p* > 0.05), *versus* Ctrl by Dunnett’s test (*right panel* only). *D* and *E*, experiment was performed concurrently with *C*, except in the presence of [U-^13^C]-glucose and assayed for glucose fermentation as outlined in the Experimental procedures. The lactate isotopologue abundance (*D*) was used to determine the absolute amount of glucose converted into lactate (*E*, *left*), which was then made relative to the Ctrl condition (*E*, *right*). Data presented as mean ± *S.D.* from 3 separate experiments, with *dot colors* matching the experiments performed in *C*. ∗, *p* < 0.05; ns, not significant (*p* > 0.05), by Dunnett's test (*right panel* only). *F*, mutuDC1940 cells were incubated 1 h with ovalbumin (Ova, 20 μg/ml) or the indicated concentrations of (intact) AIP-2 in Medium B, supplemented with 5 mM glucose, after which lactate levels were quantified by LC-MS. Data presented as mean ± *S.D.* from 4 separate experiments. ∗*p* < 0.05; ns, not significant (*p* > 0.05), *versus* Ctrl by Dunnett’s test. AIP-2, anti-inflammatory protein 2; Ctrl, control.

To achieve this, we employed MutuDC1940 cells, a cultured DC cell-line that resembles the DC subset targeted by AIP-2 *in vivo* – for instance, both exhibit retinaldehyde dehydrogenase activity (18). We measured glucose oxidation by supplementing the culture media with ^14^C-labelled glucose, measuring its incorporation into CO_2_ by our gas-trapping protocol (Fig. 2*A*). In parallel, we assessed glucose fermentation by measuring the incorporation of ^13^C-labelled glucose into lactate (11). As a control, we also treated with trypsin-digested AIP-2 (Fig. 2*B*), which does not induce immune tolerance *in vivo* (15). Interestingly, both intact and digested AIP-2 reduced glucose oxidation (Fig. 2*C*) and stimulated glucose fermentation (Fig. 2, *D* and *E*), suggesting that these metabolic changes are pleotropic to the induction of immune tolerance. To confirm that the metabolic effects of digested AIP-2 were not attributable to trace-residual intact AIP-2 remaining after trypsin exposure (Fig. 2*B*), we performed a dose curve with undigested AIP-2 spanning a 10^4^-fold range, finding that only the highest AIP-2 concentration (20 μg/ml, used in Fig. 2, *C*–*E*) increased lactate production (Fig. 2*F*). Furthermore, treatment with ovalbumin, a control protein that does not stimulate DCs in the same manner as AIP-2 (15), did not increase lactate production (Fig. 2*F*). Together, these data demonstrate that both intact and digested AIP-2 can influence DC metabolism.

Furthermore, these findings demonstrate that the optimized gas-trapping platform can quantify substrate oxidation in conventional cell culture systems. We next sought to extend the applicability of the platform to miniaturized culture formats, including organoid systems that are typically maintained in 96-well plates.

### Development of an adapter to facilitate small-scale cultures

**Figure 3:**
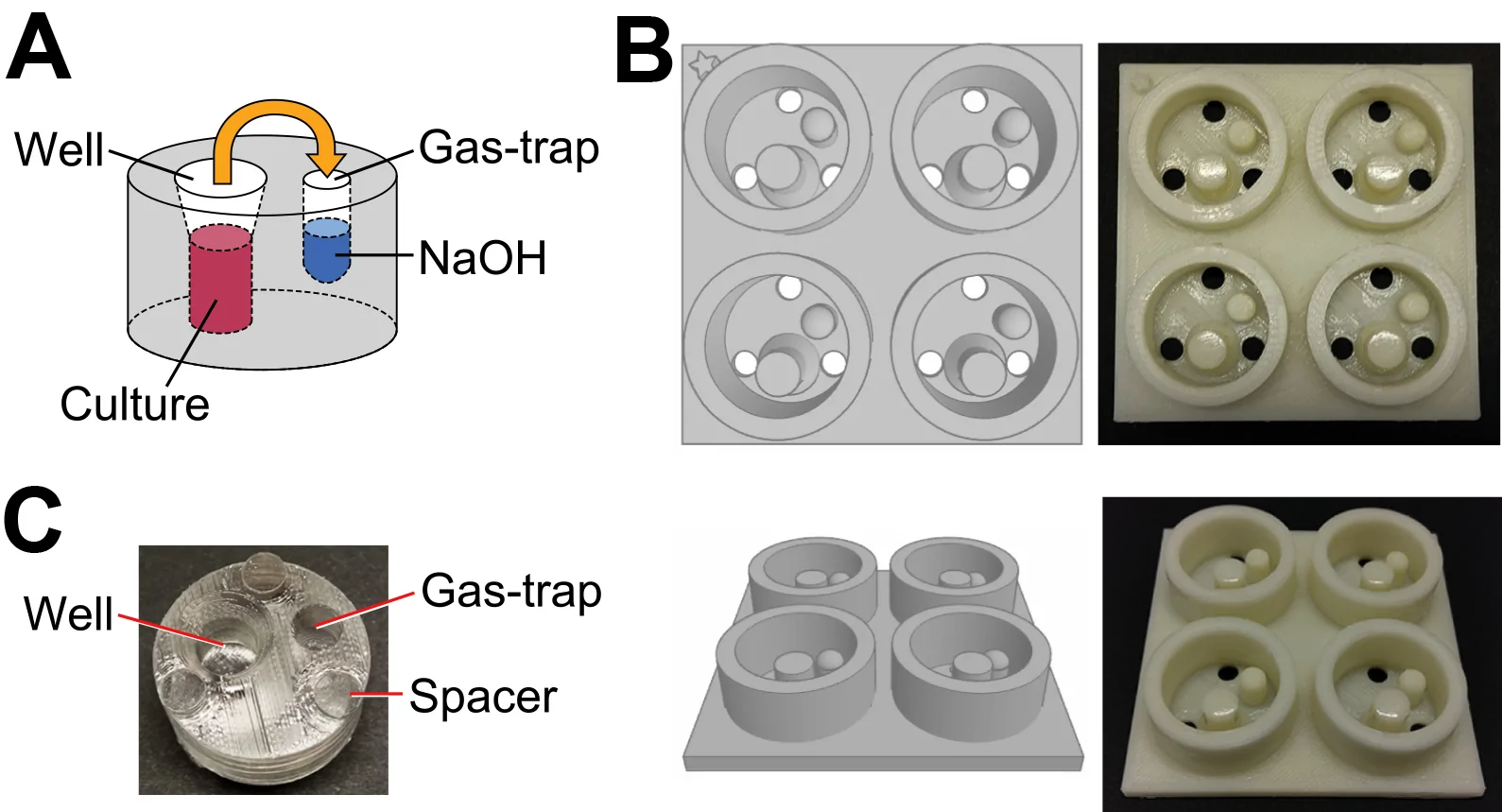
**Gas trap adapter for measuring substrate oxidation in small-scale cultures.***A*, schematic depicting the anatomy of the 12>1×96T gas trap adapter. Details in the Results. *B*, schematics and photographs depicting the mold used for casting the 12>1×96T gas trap adapters, from the top view (*top*) and side view (*bottom*). *C*, photograph depicting a typical cast using the mold in *B*.

Next, we developed an adapter that fits into a well of a 12-well plate, and housed wells for culturing cells (matching the dimensions of a single well from a 96-well plate) and holding the trapping (alkali) solution (Fig. 3*A*). We denoted this adapter as ‘12>1×96T’. The molds (Fig. 3*B*) were 3D-printed with acrylonitrile butadiene styrene (ABS), which is relatively inexpensive, durable, and can be smoothened with acetone. Using these molds, we cast adapters (Fig. 3*C*) using polydimethylsiloxane (PDMS), a silicon rubber that is biologically-inert, resistant to harsh chemicals (*e.g.*, concentrated NaOH), and has sufficient optical transparency for microscopy analysis (19). The spacers at the top of the adapters were derived from the ejection holes on the bottom of the cast (Fig. 3*B*), which enable the casts to be released from the mold using simple 3D-printed tools (Fig. S1, *C* and *D* and Table S1).

Since PDMS can absorb CO_2_, we retested the gas equilibration and trapping parameters. The previously optimized gas flow conditions (2.5 L/min for 3 min) were sufficient to equilibrate the pH of bicarbonate-buffered media (Fig. S1*E*), but the gas trapping period required extension from 1 h (Fig. 1*E*) to 24 h to capture >95% of the CO_2_ (Fig. S1*F*). Although PDMS is gas permeable, each adapter is housed within an individual well of the polystyrene tissue-culture plate, which physically separates neighboring samples and minimizes the potential for inter-well transfer of ^14^CO_2_. Thus, these PDMS adapters were compatible with our refined gas trapping protocol. We next assessed whether this platform could be applied to complex three-dimensional culture systems.

### Assessment of the metabolic response of cardiac organoids to inflammatory cytokines

**Figure 4:**
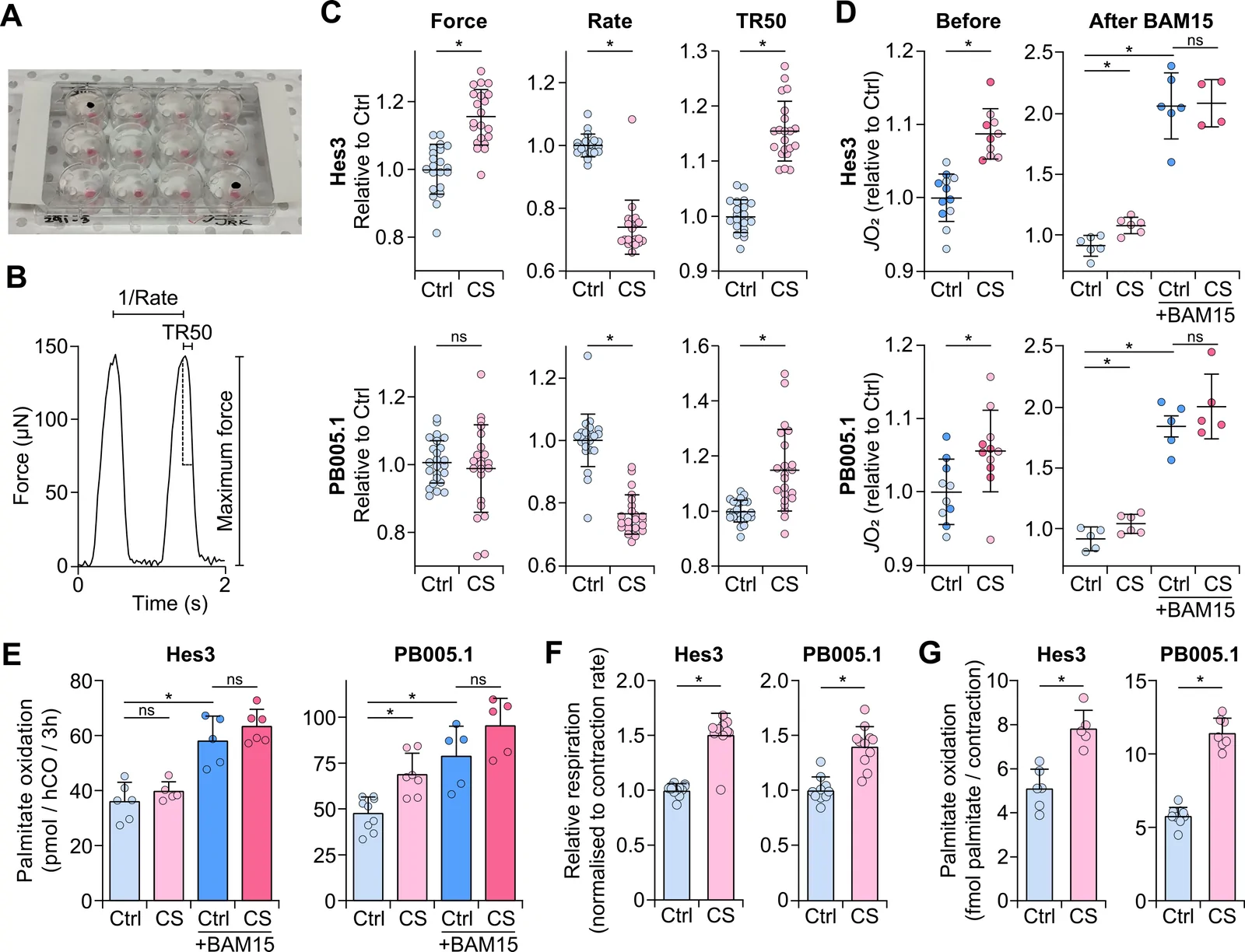
**Inflammatory cytokines do not decrease respiration or fatty acid oxidation in human cardiac organoids.***A*, photograph depicting 12>1×96T gas trap adapters installed in the 12-well plate. The plate also had Heart-Dyno chambers installed, with hCOs cultured in 150 μl of Medium E per well, with the photograph taken after sealing prior to gas equilibration. The *black dots* over the corner wells serve as a guide to ensure the manifold needles pierce the seal directly above the culture media (and hCO) in each well. *B*, typical contractile force trace for hCOs cultured within Heart-Dyno chambers in the gas trap adapters, evaluated by live cell microscopy as detailed in the Experimental procedures. *TR50*, 50% relaxation time. *C*, hCOs were treated for 24 h with cytokine storm (CS, cocktail of pI:C, IFN-γ, and IL-1β, detailed in the Experimental procedures) before contractile kinetics were evaluated as in *B*. Data presented as mean ± *S.D*. from 21 (Hes3) and 22 to 24 (PB005.1) hCOs per condition, relative to the Ctrl condition. These same hCOs were evaluated for respiration (*D*) or fatty acid-derived CO_2_ production (*E*). *D*, respiration of hCOs was measured following CS and BAM15 treatment, using the Resipher platform as outlined in the Experimental procedures. Data presented as mean ± *S.D*. from 10 to 12 (Hes3, before BAM15), 10 to 11 (PB005.1, before BAM15), 4 to 6 (Hes3, after BAM15), and 5 to 6 (PB005.1, after BAM15) hCOs per condition, relative to baseline respiration (before CS treatment) and the respiration rates of the Ctrl-treated hCOs. *E*, hCOs were cultured in the 12>1×96T gas trap adapters and treated with CS for 24 h, before being labelled with [1-^14^C]-palmitate, with or without CS and BAM15. After 3 h, fatty acid-derived CO_2_ production was determined as outlined in the Experimental procedures. Data presented as mean ± *S.D*. per hCO from 5 to 6 (Hes3) and 5 to 9 (PB005.1) hCOs per condition. *F*, relative respiration rates from (*D*) (before BAM15) were normalised to the relative contraction rate of the same hCO (rate data presented as a subset of (*C*)). Data presented as mean ± *S.D*. from 10 to 12 (Hes3) and 10 to 11 (PB005.1) hCOs per condition. *G*, fatty acid oxidation rates from (*E*) were normalized to the absolute contraction rate of the same hCO (relative contraction rate data presented as a subset of (*C*)). Data presented as mean ± *S.D*. from 5 to 6 (Hes3) and 5 to 9 (PB005.1) hCOs per condition. ∗, *p* < 0.05; ns, not significant (*p* > 0.05), by *t* test. Ctrl, control; hCO, human cardiac organoid.

We evaluated our platform using human cardiac organoids (hCOs). These are typically cultured in a 96-well plate format (19, 20), in PDMS ‘Heart-Dyno’ chambers that contain a pair of flexible poles. hCOs condense around these poles to develop spontaneous, consistent contraction. Tracking the movement of these poles with live-cell microscopy enables the assessment of contractile kinetics. To allow hCO culture in the 12-well plate adapters, heart-Dyno chamber inserts were housed in the well of the PDMS adapter (Figs. 3*C* and 4*A*). This setup was amenable to live-cell microscopy and thus the analysis of contractile kinetics (Fig. 4*B*).

It is well-known that substrate oxidation is altered in heart failure (21, 22). Pro-inflammatory conditions can alter cardiac metabolism (23) and are linked to an increased risk of heart failure (24, 25). However, it is not clear how different immunomodulatory factors influence cardiac metabolism in the absence of the immune cell effects, especially as the human heart comprises ∼5% macrophages (26). We previously recapitulated the ‘cytokine storm’ experienced in COVID-19 patients by treating hCOs with a cocktail of cytokines (interferon ɣ, interleukin 1β, poly(I:C)) in the absence of immune cells (27). hCOs responded with a slight increase in force, and substantial decrease in contractile rate and increased relaxation time (Tr50) (Fig. 4*C*). The latter is characteristic of diastolic dysfunction, which can reduce cardiac output (28). Our cytokine storm model in hCOs thus provides an opportunity to examine the impact of specific inflammatory cytokines directly on the oxidative metabolism of cardiac cells, isolated from immune cells normally present during inflammation.

Cytokine storm treatment increased baseline respiration in hCOs, but not maximal respiration following treatment with mitochondrial uncoupler, BAM15 (Fig. 4*D*) – this was observed in hCOs derived from two different human pluripotent stem cell lines, Hes3 and PB005.1. Next, we evaluated the oxidation of fatty acids, a primary substrate for the heart *in vivo* (29), by measuring the production of CO_2_ from fatty acid substrate ([1-^14^C]-palmitate). This commonly used tracer serves as a proxy for fatty acid oxidation, but it should be noted that ^14^CO_2_ recovered here reflects oxidation of the C1 carbon through β-oxidation and TCA cycle activity rather than complete molecular oxidation of palmitate *per se*. Using this tracer, fatty acid oxidation increased with cytokine treatment in hCOs derived from PB005.1 stem cells, but not Hes3 cells (Fig. 4*E*). We have recently demonstrated that substrate oxidation is driven by contraction in hCOs (14), thus we considered these metabolic parameters normalized to the contraction rate. Both respiration and fatty acid oxidation per beat increased with cytokine treatment (Fig. 4, *F* and *G*), suggesting a reduced metabolic efficiency of contraction. This demonstrates the benefits of a culture setup that permits live-cell imaging for functional analysis (Fig. 4*C*) before subjecting the same hCOs to metabolic analyses (Fig. 4, *D* and *E*). Together, our oxidation assay can be used to study the metabolism of organoids grown in small-scale culture.

## Discussion

Overall, we developed a substrate oxidation assay for small-scale *in vitro* cultures. This involved substantial optimization of our existing gas-trapping assay (Fig. 1), which we applied in conjunction with LC-MS to evaluate how a tolerogenic stimulus modulates glucose catabolism (Fig. 2). Next, we developed new adapters (Fig. 3), which enabled us to use organoid cultures to study the impact of inflammatory cytokines on cardiac fatty acid metabolism (Fig. 4). This demonstrates the utility of our assay to a broad range of two- and three-dimensional cell culture formats.

Developing a gas-trapping assay for small-scale cultures necessitated several modifications to our existing platform. This included engineering more precise control over the depth and angle of gas flow, reducing the leakiness of the manifold, and improving the adherence of the plate seals (Fig. 1). In each case, we developed cost-effective, convenient solutions, including 3D-printed components and inexpensive tools such as a glue gun, to ensure these modifications were widely accessible.

To make this assay amenable to small-scale cultures, we designed PDMS adapters that, when inserted into the 12-well plate, enabled substrate oxidation to be assayed in cells normally cultured in a 96-well plate format (Fig. 3). These can house additional inserts, such as the Dyno chamber required for culturing hCOs. Since PDMS is biologically inert, we envisage that these adapters would be well-suited for suspension and organoid cultures. While PDMS can absorb CO_2_, we elected not to apply gas-impermeable coatings because this would introduce additional fabrication complexity and impact compatibility with established hCO culture workflows (19). Instead, extending the trapping period to 24 h was sufficient to achieve >95% CO_2_ recovery (Fig. S1). Despite extending the gas-trapping period to 24 h, the plates are sealed and maintained at room temperature throughout, minimizing evaporation and background variability. We also observed that the PDMS adapters were resistant to perchloric acid-treated media. Whilst the extended trapping period requires consideration in experimental planning, it should not compromise assay integrity.

At each step of method development, we evaluated the performance of our assay platform in several ways. First, we optimized gas flow based on the equilibration of naïve media supplemented with phenol red indicator. This not only revealed that our new manifold required less time and CO_2_ for gas-equilibration, but also robust equilibration and seal adherence for all wells across the plate, both with or without the PDMS inserts. Second, we evaluated the efficiency of gas-trapping by supplementing media with ^14^C-labelled NaHCO_3_. Without a comparable method of CO_2_ capture to serve as a benchmark, this provided internal validation by serving as a proxy for cell-derived CO_2_, revealing that while 1 h is sufficient for the ‘standard’ protocol (Fig. 1), longer incubations are required when PDMS inserts are present (Fig. S1). In both cases, this assay also confirmed our improved plate-sealing protocol was suitable for gas-trapping. Last, we applied our platform to different cell-types, demonstrating its applicability to different biological contexts.

The performance of bioanalytical assays is also typically characterized by linearity and dynamic range (30). This is readily achievable when analyzing quenched samples (*e*.*g*., (18)), but can be influenced by secondary effects in live-cell assays. Using adipocyte studies as an example, the linearity of glucose oxidation rates can be evaluated by varying: (*i*) timepoints (*e*.*g*., (7)), but the impact of treatments on metabolism can vary over time when multiple substrates, metabolic equilibration, and signaling dynamics are considered (12, 31); (*ii*) cell density, but glucose partitioning in adipocytes is highly sensitive to cell density (32). Another consideration is assay precision: when considering glucose oxidation in MutuDC1940 cells (Fig. 2*C*), the intra-assay precision, assessed by the CV, was CV = 4.97% (average of *n* = 3) whilst the inter-assay precision was CV = 25.10% (*n* = 3). In contrast, the corresponding intra- and inter-assay precision were CV = 3.99% and 7.47% (*n* = 3) for the ^14^C-NaHCO_3_ recovery experiment under the same conditions (Fig. 1*F*, *1 h treatment*). The substantial difference in inter-assay precision is likely due to biological variability in basal oxidation rates, such as due to differing cell passages. This is supported by the robust AIP-2 effect observed only after normalizing to the control condition within each experiment (Fig. 2*C*, *left versus right* panels). Together, this demonstrated that evaluating the performance of live-cell assays can be complicated by secondary effects. Consequently, a key feature of our approach was adapting our gas-trapping assay to be compatible with the culture volumes and assay times previously established when typically studying our culture models – if this is not a primary motivation when establishing this protocol, such as with an uncharacterized cell culture model, then the optimization of assay uniformity (Z′ factor) would be highly recommended.

Using adherent DCs (Fig. 2), we demonstrated that AIP-2 reduced glucose oxidation with a corresponding increase in glucose fermentation. Intact AIP-2 was not required to elicit this effect (Fig. 2), yet was required to exert immune tolerance *in vivo* (15). This could reflect a pleotropic function of AIP-2, metabolically priming DCs regardless of tolerogenic effect. Treatment with metabolic modulators, for instance promoting glucose oxidation by inhibiting lactate dehydrogenase (11), would be required to confirm if this metabolic phenotype is necessary for immune tolerance. Furthermore, comparing dose curves of digested and intact AIP-2 could provide insight into how glucose fermentation is stimulated by AIP-2, for instance if digestion aids internalization or cellular signaling. Nevertheless, these data demonstrate our substrate oxidation assay can be used in conjunction with other platforms (in this case, measuring the incorporation of ^13^C-labelled glucose into lactate by LC-MS) to provide a more holistic view of cellular metabolism.

We also examined how hCOs responded to inflammatory cytokines (Fig. 4). This insult impaired contraction (Fig. 4*C*), consistent with diastolic dysfunction *in vivo*. We found that cytokine exposure increased cellular respiration (Fig. 4, *D* and *F*). Since maximal respiration was unaffected, it is likely that acute cytokine treatment does not increase substrate availability, but rather elevates respiration *via* heightened bioenergetic demand. This is supported by our substrate oxidation data, with an increase in fatty acid-derived CO_2_ production per contraction (Fig. 4*G*). This is consistent with altered coupling between fatty acid oxidation and mechanical output. We note that by using the [1-^14^C]-palmitate tracer, this endpoint specifically measures the oxidation of the labelled C1 carbon of palmitate; thus, broader conclusions about fatty acid metabolic capacity, including fatty acid uptake, (in)complete oxidation, and alternative lipid fates, require additional measurements. Further, we speculate that respiratory defects observed with heart failure (29, 33) may manifest with chronic exposure to inflammation or comorbidities such as obesity interacting to cause metabolic dysfunction, which could be explored in future studies.

Together, these experiments demonstrate that our platform can be employed to study the oxidation of individual substrates in small-scale cultures. This platform incorporates cost-effective, readily available components where possible to ensure it is broadly accessible. We have demonstrated that it is modular, making it adaptable to both a variety of cell culture formats, including 2D adherent cultures, suspension cells, and complex 3D organoid systems. It can be used in conjunction with complementary techniques such as LC-MS and live-cell imaging to provide a holistic view of cellular physiology. Overall, this platform can facilitate the study of metabolic flexibility across diverse biological systems, particularly where sample material or scale is limiting.

## Experimental procedures

### 3D printing

3D models were designed using TinkerCAD (Autodesk) and are publicly available on the Thingiverse repository (www.thingiverse.com; accession numbers in Table S1). 3D printing was performed with the 3D45 3D printer (Dremel Digilab), using either polyethylene terephthalate glycol-modified (PETG, translucent colored, 1.75 mm diameter, Dremel Digilab catalog no. PETG-TRA-01) or ABS (natural colored, 1.75 mm diameter, Aurarum catalog no. 0697478824537) filaments. Each component was printed with the material (PETG or ABS) listed in Table S1, using the printing parameters in Table S2. Further, if ABS was used: (*i*) 1% (w/v) poly(vinylpyrrolidone-co-vinyl-acetate) (aka ‘PVP-VA’) in isopropanol/water (1:1) was used as the printer bed adhesive to improve first-layer adhesion, and (*ii*) the dimensions of the models were increased by 1% during slicing to account for the shrinkage of ABS during printing.

### Construction of gas manifold

The previously designed gas manifold ((3), Table S1) consisted of a lid and base that were both printed using PETG using our updated printer settings (Table S2). The lid and base were adhered and sealed using silicon sealant (RS Components, catalog no. 555-588). Each port was fitted with a nylon male–male luer-slip connector (Cole-Parmer, catalog no. 45505-72; distributed by John Morris Scientific, catalog no. 1076298), with a 3.5 mm PETG spacer in between one end of the luer-slip connector and the manifold port (Table S1 and Fig. 1*C*). These components were adhered together with super glue (Bostik, catalog no. BO30625120), pressing the connector towards the manifold using pliers while the glue was drying, to ensure the connector and spacer were flush against the manifold base. After the glue was left to cure overnight, the other end of the connector was fitted with a beveled 18G needle (1.2 × 38 mm; Terumo TMP, catalog no. AN-1838R1). The middle needles in the first and last column were fitted with a needle guide (Figs. 1*C* and S1, *A* and *B*), which was adhered to the needle by the application of super glue to the base (hub) of the needle.

The installation of the gas manifold was as described previously (3), including: (*i*) connecting the inlet port of the manifold *via* an air-flow meter (Dwyer Instruments, catalog no. 172989-00) to a G2 gas cylinder containing 5% (v/v) CO_2_ and 21% (v/v) O_2_ in N_2_ (BOC, catalog no. LSA400011G2), and (*ii*) the lid of the manifold was heated using a 5 W heating pad (Reptile One Heat Mat 240 V, 14 × 15 cm; PetBarn, catalog no. 46527) covered with aluminum foil and monitored with an electronic temperature gauge (Jaycar Electronics, catalog no. QM7209).

### Construction of 12>1 × 96T adapters

Molds (Table S1) were printed using ABS filament. Prior to casting, the top of the central cylinder (used to cast the culture well) was smoothened with sandpaper and application of acetone. This ensured that the bottom of the well (of the casted adapter) had minimal optical interference during microscopy analysis.

The adapters were casted with PDMS (Sylgard 184, Dow Corning #761036) with a monomer:catalyst ratio of 10:1 (*w/w*). The uncured mixture was supplemented with 0.555 μl/g of platinum catalyst (platinum-divinyltetramethyldisiloxane complex in xylene, low color, 2.1-2.4% Pt, Abcr GmbH #AB153234) to prevent cure inhibition from the ABS mold. After thorough mixing, the uncured mixture was degassed for 30 min using house vacuum. During this time, the mold was prepared for casting, including removal of dust using compressed air and covering the ejection holes with plate seal (TopSeal-A PLUS, PerkinElmer, catalog no. 6050185). The uncured PDMS was poured into the mold and after a brief degassing, a paint scraper was used to remove excess PDMS, ensuring the PDMS was level with the top of the mold. After further degassing, the PDMS was cured at 65 °C for at least 35 min. After the mold was cooled to room temperature, casts were released by removing the plate seal, submerging the mold in 80% (*v/v*) EtOH, cutting casts from the edge of the mold using a microspatula, ejecting casts using 3D-printed ejection tools (Fig. S1, *C* and *D*), and allowing casts to dry overnight. Excess PDMS was trimmed using a scalpel before casts were glued into 12-well culture plates using uncured PDMS, which was allowed to cure overnight at room temperature. The casts were glued in the same orientation across the plate to ensure uniform gas equilibration during the assay.

### Cell culture

#### Materials

Pharmacological reagents used in this study include: BAM15 (MedChemExpress, #HY-110284), poly(I:C) (HMW, Invivogen), interleukin-1β (IL-1β; PeproTech #200-01B), interferon-γ (IFN-γ; PeproTech #300–02).

Recombinant AIP-2 protein (AIP-2_NTQ48_ variant, possessing a C-terminal truncation and Q48 mutation) was expressed as secreted protein using the pPICZαA vector (Invitrogen) with *Komagataella phaffii* (formerly known as *Pichia pastoris*) strain X-33 cells (Invitrogen), as described previously (15, 34). Recombinant protein was purified by nickel immobilized metal affinity chromatography with an AKTA Pure system (Cytiva), concentrated using 3 kDa cut-off centrifugal filter units (Amicon Ultra, Merck #UFC900324), and buffer exchanged into PBS. Lipopolysaccharide was removed using Triton X114 (35, 36) and confirmed to be less than 5 ng/mg by the limulus amoebocyte lysate assay (Pierce Thermo Fisher Scientific #A39553).

#### MutuDC1940

MutuDC1940 cells (37) were obtained from Applied Biological Materials (#T0528). Quality control was conducted by regular phenotypic analysis for DC identity (*e.g.*, cytokine production, capacity to induce regulatory T-cells, cell marker analysis by flow cytometry).

Cells were maintained as described previously (18). Briefly, cells were cultured in Medium A, consisting of RPMI1640 (#61870127, with GlutaMAX supplement) supplemented with 10% (*v*/*v*) fetal bovine serum (#10099141), 10 mM Hepes (pH 7.4, #15630080), 55 μM β-mercaptoethanol (#21985023), and 100 U/ml penicillin/streptomycin (#15140122), all sourced from Thermo Fisher Scientific.

#### hCOs

Ethical approval for the use of human embryonic stem cells was obtained from QIMR Berghofer’s Ethics Committee and carried out in accordance with the National Health and Medical Research Council of Australia (NHMRC) regulations, abiding by the Declaration of Helsinki principles. Human embryonic stem cells utilized were HES3 (WiCell, RRID: CVCL_7158). Informed consent was obtained from all participants for the derivation and use of induced pluripotent stem cells including PB005.1 (female) (38) from Murdoch Children’s Research Institute. Quality control was performed by short tandem repeat profiling, karyotyping and *mycoplasma* testing.

Cardiac cells were generated from human pluripotent stem cells using a single-step differentiation protocol described previously (39), after which hCOs were generated in Heart-Dyno chambers using the ‘directed maturation’ protocol described previously (40). Following the final incubation with DY131 and MK8722, hCOs were washed and refreshed with Medium C, defined previously as ‘weaning medium’ (27, 40). Two days later, hCOs were exposed to inflammatory stress by refreshing with weaning medium supplemented with cytokines (10 μg/ml poly(I:C), 10 ng/ml IL-1β, and 100 ng/ml IFN-γ) for 24 h (27).

In line with ISSCR guidelines (https://www.isscr.org/basic-research-standards), data panels depicting hCO data present each hCO as an individual data-point. Further, data from hCOs derived from different cell-lines are shown on separate panels.

#### Digestion of AIP-2

To digest AIP-2, 2 mg/ml of AIP-2 was incubated for 1 h at 37 °C with 2 mg/ml trypsin (no phenol red, Thermo Fisher Scientific #15090046) in PBS. An aliquot was mixed with denaturing PAGE sample buffer (NuPage LDS sample buffer, Thermo Fisher Scientific #NP0007) and TCEP (Thermo Fisher Scientific #77720), boiled at 60 °C for 10 min, and subjected to SDS-PAGE and Coomassie staining to confirm the digestion of AIP-2. The remainder was quenched by a 10-fold dilution with Medium B, resulting in a 10× solution of AIP-2 that achieves a final concentration of 20 μg/ml. As controls, PBS (vehicle), AIP-2 alone, and trypsin alone were incubated in parallel under the same conditions.

### Metabolic labelling

In each experiment below, the assay media was allowed to preequilibrate at 37 °C and 5% (*v/v*) CO_2_ for at least 30 min beforehand.

#### MutuDC1940

Adherent cells were lifted with PBS at 37 °C and resuspended in Medium B, consisting of glucose-free RPMI1640 (Thermo Fisher Scientific #11879020) supplemented with GlutaMAX, 10 mM Hepes, 55 μM β-mercaptoethanol, 100 U/ml penicillin/streptomycin, and 0.5% (*w*/*v*) fatty acid-free bovine serum albumin (Sigma-Aldrich #A7030). Medium B is identical to Medium A except the glucose has been omitted and serum replaced with albumin. Following counting, cells were resuspended in Medium B at 1.25 million cells/ml. 0.4 ml of cell suspension (0.5 million cells) was dispensed into each well of a 12-well plate, followed by the addition of 50 μl of 10 × solution of AIP-2 (intact or trypsin-digested, prepared as described above) and 50 μl of labelled glucose. The latter yielded final concentrations of (*i*) 5 mM glucose and 0.5 μCi/ml [U-^14^C]-glucose (PerkinElmer #NEC042X) for the substrate oxidation assay, or (*ii*) 5 mM [U-^13^C]-glucose (Cambridge Isotope Laboratories #CLM-1396) for the lactate production assay. This permitted two separate assays (on separate plates) to be performed in parallel from the same batch of cells.

#### hCOs

hCOs were first washed with 150 μl/well of Medium D, which was identical to Medium C except supplemented with (*i*) 15 mg/L phenol red (Sigma-Aldrich #P5530) to monitor pH during gas equilibration, (*ii*) 0.5 mM glutamine as a more readily-available source of this nutrient in addition to GlutaMAX, (*iii*) 0.4 mM lactate and 0.1 mM pyruvate, based on their steady state levels when hCOs are cultured in Medium C (unpublished observations), (*iv*) without glucose, and (*v*) 100 μM palmitate, conjugated to the albumin in the B27 supplement.

hCOs were then incubated in 120 μl/well of Medium E, which was consisted of Medium D supplemented with (*i*) 5.5 mM glucose, providing this nutrient at a physiologically relevant concentration, and (*ii*) 0.5 μCi/ml [1-^14^C]-palmitic acid (PerkinElmer #NEC075H), which was conjugated alongside the 100 μM palmitate to the albumin in the B27 supplement. This was followed by the immediate addition of 30 μl of 5 × solution of (*i*) cytokines, to achieve final concentrations of 10 μg/ml poly(I:C), 10 ng/ml IL-1β, and 100 ng/ml IFN-γ, and/or (*ii*) BAM15, to achieve a final concentration of 10 μM.

#### Gas-phase equilibration of cell culture microplates

The heating pad was switched on at least 30 min prior to experiments. After media was dispensed into the cell culture microplate, the plate was sealed as described previously (3), with modifications. To improve the adherence of the plate seal, we used hot glue dispensed using the glue gun 930 (Dremel, catalog no. F0130930NA), on the low-temperature setting (105 °C) to avoid melting the microplate plastic. Using 7 mm clear, low-temperature glue sticks (GG02, Dremel, catalog no. 2615GG02JA), hot glue was deposited in each corner of the plate deck (the top, flat surface surrounding the wells, within the plate rim). A plate seal (TopSeal-A PLUS, PerkinElmer, catalog no. 6050185) was immediately applied and the glue was made level to the well rim by applying a 150 mm plastic scraper (Pro Renovator, Bunnings catalog no. 1610000) in a motion parallel to the short side of the plate.

After the glue was set, a hole was made in each well using a beveled 18G needle, aimed off-center preferably where the NaOH would later be dispensed. A 78 mm plastic scraper (3D-printed, Table S1) was then applied in a motion parallel to the long side of the plate to ensure the plate seal remained adhered to the plate. The plate was placed on a laboratory jack (Eisco 150 × 135 mm; WestLab, catalog no. 071210-0001) and lifted up to the gas manifold until the manifold needles were inserted to the depth specified by the needle guide. The needles either punctured the center of the well, either of the 12-well plate (with no adapter) or within the adapter itself. We found this was best achieved by marking the desired location on the corner wells (Fig. 4*A*, black dots on plate seal). The gas phase in each well was then equilibrated at 2.5 L/min for 3 min, unless otherwise specified in the figure legends. After equilibration, the plate was removed from the manifold and another layer of plate seal was immediately applied using the 78 mm plastic scraper. The plate was incubated as described in the figure legends.

#### Substrate oxidation assay

Substrate oxidation was measured by CO_2_ trapping, as described previously (3) for the protocol without an adapter except with the updated gas equilibration methodology outlined above. For performing the assay with the 12>1×96T adapter present, 100 μl of 5 M NaOH was dispensed *via* a beveled 27G needle (0.4 × 13 mm; Terumo TMP, catalog no. NN-2713R) into the gas trap, and 100 μl of 3 M perchloric acid *via* a beveled 21G needle (0.8 × 38 mm; catalog no. above) to the culture medium. The plate was immediately resealed using TopSeal-A PLUS and left at room temperature for 24 h. The radioactivity of the gas-trapping solution (NaOH) was measured by transferring the solution to 3 ml of Ultima Gold XR scintillant (PerkinElmer, catalog no. 6013119) and performing liquid scintillation counting using the TriCarb 4910TR (PerkinElmer). Radioactivity of samples were normalized to cell-free controls (*i.e.*, cell-independent signal) assayed in parallel and the substrate specific activity (3) to obtain the absolute conversion of substrate into CO_2_.

### Evaluation of medium pH following gas equilibration

Medium pH was determined using phenol red, measuring the absorbance at 430 and 560 nm. We previously demonstrated that the A560/A430 ratio correlated with pH between pH 6.5 and 9.5 (3). This approach is advantageous over conventional methods (*e.g.*, by pH meter) since it requires only a small sample volume and prevents prolonged exposure to air.

Culture media (DMEM with 15 mg/L phenol red) was preequilibrated at 5% CO_2_ and then dispensed with the typical assay volume: 500 μl/well in 12-well plates without an adapter or 150 μl with 12>1×96T adapters present. In the former format (Fig. 1), the time taken for installing the gas traps may result in media alkalinization, which we simulated by exposure to air for at least 10 min. We skipped this step for the latter format (Fig. 3) since the gas trap is embedded within the 12>1 × 96T adapter. Plates were then sealed and gas-equilibrated with the gas manifold at the indicated flow-rates and durations. Following equilibration, plates were sealed and incubated for at least 1 h at 37 °C to simulate experimental conditions. Using 21G needles, media was transferred to microfuge tubes ensuring minimal air volume within tubes. One sample at a time, 100 μl was dispensed into a 96-well plate and phenol red absorbance was immediately measured. The A560/A430 ratio was converted into media pH using a standard curve generated by titrating naïve media to a range of pH’s using NaOH and HCl, as done previously (3).

We previously measured absorbance directly in the 12-well plate (3), but our new approach: (*i*) increases the pathlength, reducing errors in spectrophotometry, (*ii*) reduces the surface area exposed to air and thus risk of alkalinization, (*iii*) provides a stopping point, and (*iv*) is compatible with evaluating media pH in the 12>1×96T adapters.

### Evaluation of plate seal adherence

Plate seal adherence was evaluated after sealing 12-well plates with or without hot glue applied to the corners. This was achieved using 0.008% (w/v) Trypan Blue, prepared by diluting 0.4% Trypan Blue (Thermo Fisher Scientific #T10282) with water, to enable visual inspection for leaks.

#### *Inversiontest*

0.5 ml of diluted Trypan Blue solution was dispensed into each well. Plates were sealed (with or without hot glue), then the seal was punctured above each well with an 18G needle before being resealed, to simulate gas equilibration. Plates were inverted and gently swirled to ensure the Trypan Blue solution was in contact where the well was adhered to the plate seal. Plates were left at room temperature overnight before being inspected for leaks.

#### Air injection test

Plates were prepared and sealed as with the inversion test, except the diluted Trypan Blue solution was only dispensed into the four corner wells. For two opposing corner wells, a drop of hot glue was applied directly onto the plate seal, above the center of the well, before an 18G beveled needle was immediately pierced through the glue and seal. As the glue set, this created an air-tight seal between the needle and seal. A 10 ml syringe was filled with air and gently inserted into the needle. The plate was inserted and air was slowly injected into the well until seal delamination occurred. This procedure was repeated for the opposing corner well, providing two replicate measurements for each plate. The remaining two corner wells served as controls.

### Quantification of lactate

The conversion of glucose into lactate was measured using the approach described previously (11), with modifications described below. Culture media was extracted for polar metabolites using 4 vol of ACN/MeOH (*v/v* 1:1) with 1 μM morpholineethanesulfonic acid as an internal standard, as described previously (41). Calibration standards were diluted into naïve media and extracted in parallel to the experimental samples.

Following extraction, polar metabolites were subjected to LC as described previously (41), except modified to achieve a rapid assay for lactate quantification with a run-time of 11 min (*versus* 18.5 min for the original method). This was achieved using a 1290 Infinity II pump (Agilent) with an InfinityLab Poroshell 120 HILIC-Z column (Agilent; 100 mm length × 2.1 mm internal diameter, 2.7 μm particle size, stainless steel with compatible guard column attached). Before use, the LC system was passivated by washing overnight with 0.5% (*v/v*) phosphoric acid in ACN/H_2_O (90:10) (41). LC separation was achieved using Buffer A, consisting of ACN/H_2_O (10:90) with 10 mM ammonium acetate (set to pH 9 with NH_4_OH) and 5 μM medronic acid, and Buffer B, consisting of ACN/H_2_O (80:20) with the same additives as Buffer A. The LC gradient was as follows: 0 min, 100% A, 0.25 ml/min; 2 min, 100% A, 0.25 ml/min; 4 min, 90% A, 0.25 ml/min; 4.5 min, 60% A, 0.25 ml/min; 6.5 min, 60% A, 0.25 ml/min; 7 min, 100%, 0.5 ml/min; 10.9 min, 100%, 0.5 ml/min; 11 min, 100%, 0.25 ml/min. The autosampler temperature was 4 °C, column temperature was 30 °C, and injection volume was 3 μl.

Resolved metabolites were analyzed by MS using an Agilent 6470 QQQ with Jet Stream Technology Ion Source, with parameters as defined previously (41). Optimized transition parameters are listed in Table S3.

LC-MS data were extracted using Skyline software (42) and the absolute quantification of target metabolites was achieved using the calibration standards. Lactate ^13^C-isotopologue levels were corrected for natural abundance using the IsoCor package (43). This was used to calculate the abundance of ^13^C-labelled lactate, being the product of ^13^C-fractional contribution and total abundance of lactate (11, 31). Since one glucose molecule generates two lactate molecules, the ^13^C-labelled lactate abundance was halved to determine the conversion rate of glucose into lactate.

### Respirometry

Live-cell respiration was measured using the Resipher platform (Lucid Scientific), as described previously (40) with minor modifications. Briefly, hCO were cultured in 96-well microplates (Nunc MicroWell, Thermo Fisher Scientific catalog #167008), using Heart-Dyno chambers with the height reduced to 1.5 mm to avoid collision with the Resipher sensor probes. The Resipher sensor lid with 9.4 mm probe length (Lucid Scientific catalog #NS32-94A) was utilized with an operating height between 1600 and 2100 μm. In each experiment, adjacent empty wells were filled with naïve media to minimize evaporation and cell-free control wells were included in each column of wells analyzed by the Resipher sensor lid.

At least 24 h prior to respirometry analysis, the Resipher sensor lid was preequilibrated with naïve media in a cell-free 96-well microplate at 37 °C and 5% (*v/v*) CO_2_. The Resipher sensor lid was stored under similar conditions during media changes and microscopy analysis. The experiment was conducted in a dedicated tissue culture incubator to minimize environmental fluctuations, such as changes in humidity, temperature, and vibrations from opening the incubator door.

On Day 28 after the initiation of cardiac cell differentiation from human pluripotent stem cells (13 days after hCO formation), the hCOs were refreshed with 150 μl culture media per well and the Resipher sensor lid was transferred to the hCO culture plate. This was 2 days prior to treatments, to permit quality-control checks such as stability in respiration rates and to establish baseline respiration rates prior to treatment. Treatment involved refreshing hCOs with 150 μl of media supplemented with or without cytokines, as outlined above. After 24 h, hCO were treated with or without 10 μM BAM15. This was achieved by the addition of 10 μl of media, containing 16x BAM15 or DMSO (vehicle control), instead of a complete media refresh to minimize equilibration time (approximately 6 h). For each time point, respiration rates were normalized to cell-free controls and made relative to the baseline respiration rate for each respective hCO.

### Functional analysis of hCOs

Contractile analysis of hCOs was performed using live cell microscopy, as described previously (27), using the Leica Thunder microscope. Briefly, hCOs were imaged at 37 °C and 5% (*v/v*) CO_2_ over 10 s (500 frames). Videos were obtained before (baseline) and after treatments, and analyzed using in-house MATLAB scripts (19). Functional parameters were normalized to the baseline values and to the average of control-treated hCOs.

## Data availability

STL files used for 3D-printing are publicly available on Thingiverse (accession numbers provided in Table S1). Unless otherwise indicated, the data described here is contained within the manuscript. Unpublished data can be shared upon request by contacting the corresponding authors (J. R. K., james.krycer@qimrb.edu.au; S. N., severine.navarro@qimrb.edu.au).

## Supporting information

This article contains supporting information.

## Conflicts of interest

J. E. H. and R. J. M. are co-inventors on a patent relating to the Heart-Dyno device and human cardiac organoid maturation (WO2018035574A1 filed by the University of Queensland), which is licensed to Dynomics. J. E. H. is a co-inventor on a provisional patent filed by QIMR Berghofer on new cardiac maturation conditions (2024902826). J. E. H. and R. J. M. are co-inventors on a patent for cardiac regeneration therapeutics (WO2020186283A1 filed by QIMR Berghofer). J. E. H. is co-inventor on a patent for the serum-free conditions supporting the vascular population used in this study (WO2024016058 filed by MCRI and QIMR Berghofer). J. E. H. is co-inventor on licensed patents for cardiac differentiation and engineered heart muscle (WO2015040142A1 and WO2015025030A1), which are licensed to MyriaMed and Repairon. J. E. H. and R. J. M. are co-founders, scientific advisors, and stockholders in Dynomics. S. N.’s research is supported by the CSL Research Accelerator Initiative. The remaining authors declare no competing interests.
