# Supplementary material for: A modular platform for measuring substrate oxidation by CO2 production in diverse cell culture systems
Source: J Biol Chem. 2026 Aug 4;302(9):113384. doi: 10.1016/j.jbc.2026.113384 (PMC13559768; doi:10.1016/j.jbc.2026.113384)
Supplement: Supplementary file 1 [file mmc1.docx]

**A modular platform for measuring substrate oxidation by CO_2_ production in diverse cell culture systems**

**SUPPORTING INFORMATION**

James R. Krycer^†^*, Shubhangi Seth^†^, Lynn A. C. Devilée, Mary Lor, Rebecca L. Fitzsimmons, Richard J. Mills, Yan Lu, Prarthna G. Bhaskaran, Mark Hodson, James E. Hudson, Severine Navarro*

^†^ Equal contribution

* Corresponding authors

**Contents:**

Figure S1

**Figure S1. Supplemental data for Fig. 1 and 3. *A*-*B*,** Schematic (**A**) and photograph (**B**) of the needle guide installed in the gas manifold (**Fig. 1C**). In (**B**), an 18G needle has been inserted into the guide. ***C***-***D***, Schematic (**C**) and photograph (**D**) of the ejection tools used to eject the casts of the 12>1x96T adapters (**Fig. 3C**) from the mold (**Fig. 3B**). ***E***, Culture media was equilibrated in the 12>1x96T adapters using the gas manifold at a flow rate of 2.5 L/min for the indicated durations, and pH was measured by phenol red absorbance, as described in the *Experimental Procedures*. Data presented as mean ± *S.D.* from three separate experiments. ***F***, The experiment in **Fig. 1F** was repeated with the 12>1x96T adapters. Data from individual experiments are shown.
